# A qualitative analysis to identify implementation strategies for a deprescribing clinical practice guideline using the CFIR-ERIC Matching Tool: perspectives from consumers and professional stakeholders

**DOI:** 10.1007/s11096-026-02218-9

**Published:** 2026-09-03

**Authors:** Hui Wen Quek, Xisco Reus, Kenneth Lee, Veronika Seda, Leon Adams, Osvaldo P. Almeida, Gillian E. Caughey, Jennifer Gowan, Kerryn Hart, Andrew Heredia, Anne-Marie Hill, Lisa Kalisch Ellett, Dina LoGiudice, Arduino A. Mangoni, Anthony Marinucci, Treasure McGuire, Nilufeur McKay, Vasi Naganathan, Christopher Ng, Maz Osborne, Kevan R. Polkinghorne, Constance D. Pond, Sepehr Shakib, Angela Shiu, Andrew Stafford, Tim Whitmore, Christopher Etherton-Beer, Amy Page

**Affiliations:** 1 Centre for Optimisation of Medicines, School of Health and Clinical Sciences, The University of Western Australia, 35 Stirling Highway, Crawley, Perth, WA 6009 Australia; 2 Department for Health and Wellbeing, Adelaide, Australia; 3 The University of Sydney, Sydney, Australia; 4 Sir Charles Gairdner Hospital, Perth, Australia; 5 The University of Western Australia, Perth, Australia; 6 Western Australian Centre for Health and Ageing, The University of Western Australia, Perth, Australia; 7 Institute for Health Research, The University of Notre Dame Australia, Fremantle, Australia; 8 Registry of Senior Australians Research Centre, South Australian Health and Medical Research Institute, Adelaide, Australia; 9 Flinders University, Adelaide, Australia; 10 Gowan & Associates Pty Ltd, Melbourne, Australia; 11 Monash University, Melbourne, Australia; 12 School of Medicine (Optometry), Deakin University, Waurn Ponds, Australia; 13 Australian Dental Association Victorian Branch, Melbourne, Australia; 14 Quality Use of Medicines and Pharmacy Research Centre, Clinical and Health Sciences, The University of South Australia, Adelaide, Australia; 15 The Royal Melbourne Hospital, Melbourne, Australia; 16 The University of Melbourne, Melbourne, Australia; 17 Victorian Aboriginal Health Service, Melbourne, Australia; 18 Southern Adelaide Local Health Network, Adelaide, Australia; 19 Royal Australian College of General Practitioners, Melbourne, Australia; 20 School of Pharmacy and Pharmaceutical Sciences, The University of Queensland, Brisbane, Australia; 21 Faculty of Health Sciences and Medicine, Bond University, Gold Coast, Australia; 22 Mater Health SEQ, South Brisbane, Australia; 23 School of Nursing and Midwifery, Edith Cowan University, Joondalup, Australia; 24 Royal Perth Hospital, Perth, Australia; 25 Centre for Education and Research on Ageing, Department of Geriatric Medicine, Concord Hospital and Concord Clinical School, Sydney, Australia; 26 Faculty of Medicine and Health, The University of Sydney, Sydney, Australia; 27 Blacktown Hospital, Sydney, Australia; 28 Consumer/Carer Representative, Perth, Australia; 29 Department of Nephrology, Monash Health, Melbourne, Australia; 30 Department of Medicine, Monash University, Melbourne, Australia; 31 Wicking Dementia Research and Training Centre, The University of Tasmania, Hobart, Australia; 32 Northern Adelaide Local Health Network, Adelaide, Australia; 33 Adelaide University, Adelaide, Australia; 34 Advanced Pharmacy Australia, Melbourne, Australia; 35 Austin Health, Better@Home Subacute Program, Melbourne, Australia; 36 Curtin Medical School and enAble Institute, Curtin University, Perth, Australia

**Keywords:** Aged, Deprescriptions, Implementation science, Polypharmacy, Primary health care, Qualitative research

## Abstract

**Introduction:**

Deprescribing is the planned withdrawal or dose reduction of medicines when harms outweigh benefits or when they are no longer required. Following publication of the Clinical Practice Guideline for Deprescribing in Older People in September 2025, supporting its uptake into routine practice is a critical next step.

**Aim:**

This study aimed to identify implementation strategies, informed by stakeholder-perceived barriers, to support the implementation of deprescribing guidelines.

**Method:**

A qualitative analysis was undertaken of open-text responses collected during the guideline public consultation (24 April–31 May 2025). Inductive thematic analysis identified implementation barriers, which were then deductively mapped to the Consolidated Framework for Implementation Research (CFIR). Relevant CFIR constructs were entered into the CFIR-Expert Recommendations for Implementing Change (ERIC) Matching Tool to generate implementation strategies. Strategies were ranked by cumulative percentage endorsement and grouped using ERIC concept-mapping clusters.

**Results:**

Of 313 survey respondents, 248 provided responses relevant to implementation. Three barrier themes were shared across consumer and professional/policy stakeholder groups. Theme 1: ‘Knowledge gaps, beliefs, and limited readiness to deprescribe’ describes gaps in consumers’ and clinicians’ knowledge, beliefs about medication use and deprescribing, and limited confidence or willingness to engage in deprescribing. Theme 2: ‘Communication, hierarchy, or social influences limiting deprescribing’ describes communication gaps, entrenched prescribing norms, power imbalances, and social influences that limited shared decision-making. Theme 3: ‘System-level structures misaligned with deprescribing practice’ describes broader healthcare system barriers, including financial, policy, workforce, organisational, and information-related constraints. Consumers additionally highlighted a lack of person-centred and adaptable approach for deprescribing, while professional/policy stakeholders identified barriers related to medicine formulations and guideline format and usability. Barriers mapped predominantly to the CFIR Inner Setting domain. The highest-ranked implementation strategy was ‘Identify and prepare champions’, within the ‘Develop stakeholder interrelationships’ cluster.

**Conclusion:**

Deprescribing barriers were largely situated within the Inner Setting domains. Recommended strategies emphasised stakeholder engagement and local champions, supporting locally led, relationship-based implementation approaches. Future work should evaluate the feasibility, acceptability, and effectiveness of these strategies in practice.

**Supplementary Information:**

The online version contains supplementary material available at https://doi.org/10.1007/s11096-026-02218-9.

## Impact statements

- This study highlights that deprescribing requires supportive practice environments that enable effective communication, collaboration, and shared decision-making between consumers and clinicians.
- The findings identify key implementation strategies, including local champions, stakeholder partnerships, and context-adapted approaches, to support healthcare professionals in integrating deprescribing into routine practice
- Strengthening consumer-clinician partnerships may empower consumers to actively participate in medication decisions, support pharmacists and clinicians in delivering person-centred medication optimisation, and improve the sustainability of deprescribing practices across healthcare settings.

## Introduction

Deprescribing involves the withdrawal or dose reduction of medicines when potential harms outweigh benefits, or when they are no longer required [1, 2]. Emerging evidence demonstrates clinically meaningful benefits, including reduced mortality, when patient-specific interventions are initiated early [3]. To support clinical decision-making, an evidence-based Clinical Practice Guideline for Deprescribing in Older People (the guideline) was published [4, 5].

Deprescribing is particularly relevant in primary care, where most prescribing, dispensing, and medication review occur [6–8]. A recent study using routine dispensing data revealed a high prevalence of potentially inappropriate medicine use in older people, highlighting opportunities for deprescribing [9]. Multidisciplinary collaboration involving GPs, pharmacists, nurses and other primary care professionals is increasingly recognised as important for medicine optimisation. With the expanding scope of pharmacist practice, including medication reviews and prescribing responsibilities in some jurisdictions [10], pharmacists are increasingly positioned to contribute to deprescribing and broader medicines optimisation initiatives. Studies have shown that GP-pharmacist collaboration in primary care settings can reduce the use of potentially inappropriate medicines [11, 12]. However, pharmacists face structural and competency-related barriers to collaborative deprescribing [13].

While guidelines facilitate the translation of evidence-based recommendations into routine care, sustained guideline implementation requires careful consideration of service delivery and the contextual factors that influence how guidelines are perceived, adopted, and applied in practice. In implementation science, these contextual factors are commonly referred to as determinants, encompassing both barriers and enablers [14, 15]. Determinants help explain why interventions achieve intended outcomes in some contexts but not others [16, 17]. Implementation science frameworks provide structured approaches for identifying determinants and guiding translation into practice [15]. The Consolidated Framework for Implementation Research (CFIR) [18, 19] is a widely used determinant framework that categorises factors influencing implementation across five domains: Intervention characteristics, Outer setting, Inner setting, Characteristics of individuals, and Process [18, 19]. The CFIR-Expert Recommendations for Implementing Change (CFIR-ERIC) Matching Tool [20–22] was later developed to identify targeted implementation strategies that will best overcome each CFIR barrier. It comprises 73 strategies ranked by their relevance to addressing barriers and has been applied across healthcare implementation contexts [23].

Recent primary care research exploring determinants of deprescribing implementation using CFIR has identified determinants related to intervention characteristics, compatibility with clinical workflows, healthcare professionals’ knowledge and beliefs, patient needs and engagement, and organisational readiness for implementation [24]. However, targeted theory-informed implementation strategies to address these barriers remain limited. This study addresses this gap by applying the CFIR‑ERIC Matching Tool to stakeholder-identified barriers to generate targeted strategies for implementation.

### Aim

This study aimed to identify strategies, informed by perceived barriers, to support the implementation of deprescribing guidelines across consumers, community members, clinicians, and the broader healthcare system.

## Method

### Study design and context

This study presents a qualitative analysis of open-text responses prospectively collected through the guideline public consultation survey conducted from 24 April to 31 May 2025, as part of the guideline development process (https://deprescribing.com/about/documents-for-public-consultation.html) (Supplementary File 1) [5]. The consultation followed the National Health and Medical Research Council standards for guideline development [25] to invite stakeholder feedback on draft recommendations before finalisation. Participation was voluntary, with purposive, convenience, and snowball sampling used. Purposive recruitment targeted consumers and community members with medication management experience, as well as stakeholders with relevant expertise or interest in deprescribing. Convenience sampling was facilitated through media and web-based invitations. A response rate could not be calculated due to multiple recruitment channels. No saturation threshold was applied, as the aim was to maximise stakeholder feedback. The study was underpinned by an interpretivist or constructivist research paradigm [26] and reported according to the Standards for Reporting Qualitative Research (SRQR) to ensure rigour and transparency (Supplementary File 2, Table S1) [27]. Although the consultation captured broad stakeholder perspectives, consumer responses were interpreted in the context of their healthcare experiences, particularly primary care where most medicine management occurs [8].

### Questionnaire development and administration

The public consultation questionnaire was administered using Qualtrics (Supplementary File 3). The present study analysed and reported open-ended responses specific to implementation questions only. Three optional questions explored reflections on deprescribing guideline implementation by stakeholder type (Table 1). These questions were developed iteratively by the guideline steering committee to elicit feedback on clarity, relevance, utility, and anticipated barriers/facilitators to implementation. Plain language principles, including the use of short sentences, avoidance of medical jargon, and familiar, everyday terminology, were used to enhance accessibility and support consumer engagement. No modifications were made to the questionnaire during data collection. The questionnaire was not validated or pilot-tested due to time constraints. The full consultation report is available elsewhere (https://deprescribing.com/) [5].

**Table 1:** Stakeholders and corresponding question sets

Stakeholders	Question sets
Consumers and community members	1. Would you require any additional support to discuss deprescribing with your healthcare providers? 2. What barriers do you think you would encounter in discussing deprescribing with your healthcare providers? 3. What makes it easier to discuss deprescribing with your healthcare providers?
Professional and policy stakeholders	1. Would you require any additional resources or tools to implement this guideline in practice? 2. What barriers do you think people would encounter in implementing the guideline? 3. Are there any facilitators of using this guideline in clinical practice?

The questionnaire used branching logic to present separate question sets for (i) consumers and community members and (ii) professional/policy stakeholders, based on self-reported role or interest in deprescribing (Table 1). Participants who identified with both roles were shown both question sets. Consumers and community members included those with medicine management experience, carers or relatives of long-term medicine users, consumer advocates, or interested members of the public. Professional and policy stakeholders included healthcare professionals, health policymakers, service providers, and researchers/academics with a work-related interest in deprescribing. The questionnaire explored guideline implementation determinants among professional and policy stakeholders and consumer-level support needs, barriers, and facilitators for engaging in deprescribing with healthcare providers.

### Researcher characteristics

The coding team comprised a pharmacist (HWQ; female), a GP (XR; male), and a consultant pharmacist (AP; female) with prior experience in deprescribing and guideline development. This may have sensitised interpretation toward primary care and aged care implementation issues. To address this, the team maintained an audit trail of analytic decisions, conducted independent coding with reconciliation, and held meetings to discuss assumptions and alternative interpretations. Existing networks were encouraged to participate, with potential influence from prior researcher-participant relationships addressed through content-focused analysis, reflexive discussions, and structured review processes.

### Data analysis

**Fig. 1:**
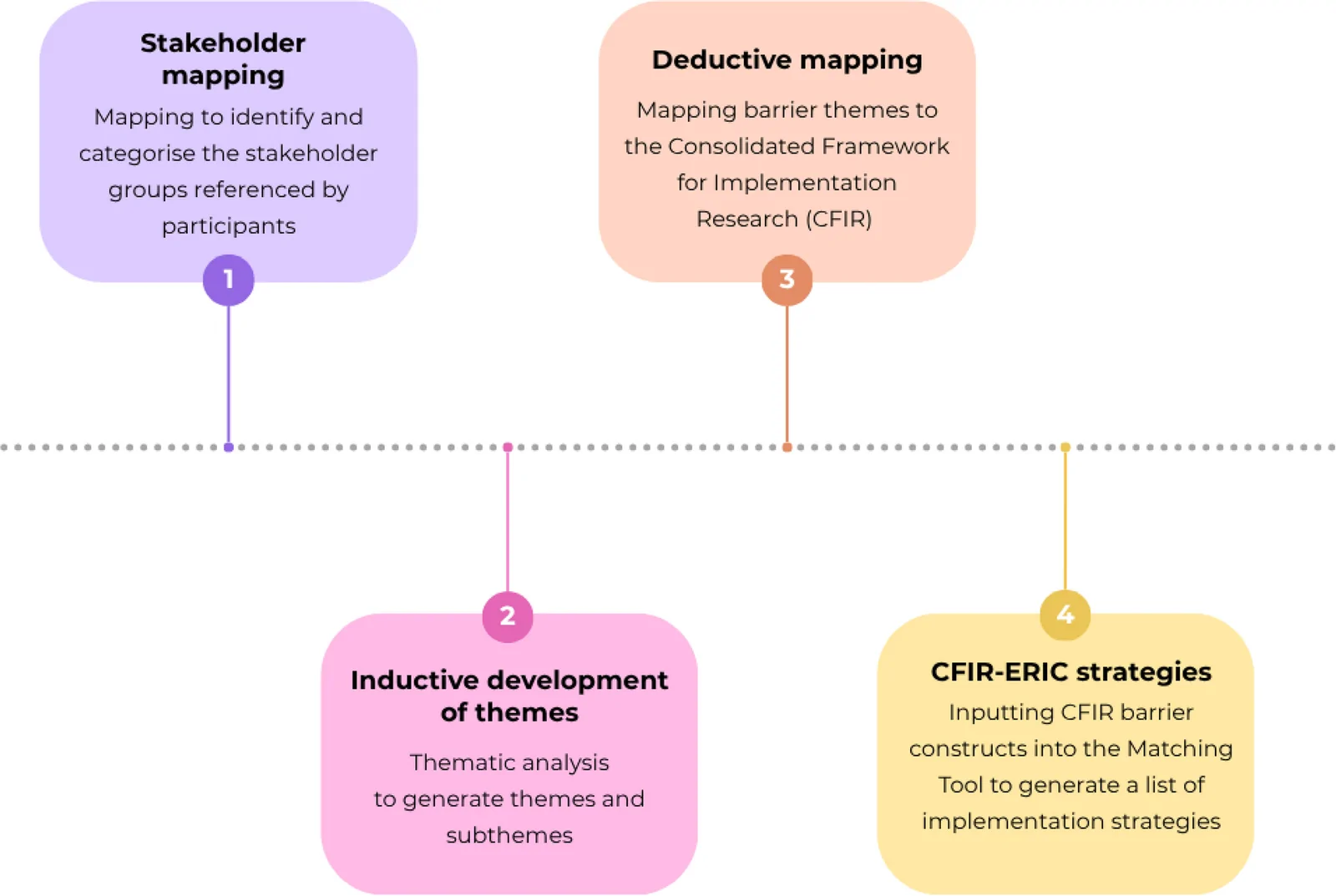
Overview of the steps involved in the data analysis

Data were exported from Qualtrics to Microsoft Excel® (Version 16.104.1). A four-phase sequential analysis was undertaken (Fig. 1).

### Phase I (stakeholder mapping)

**Fig. 2:**
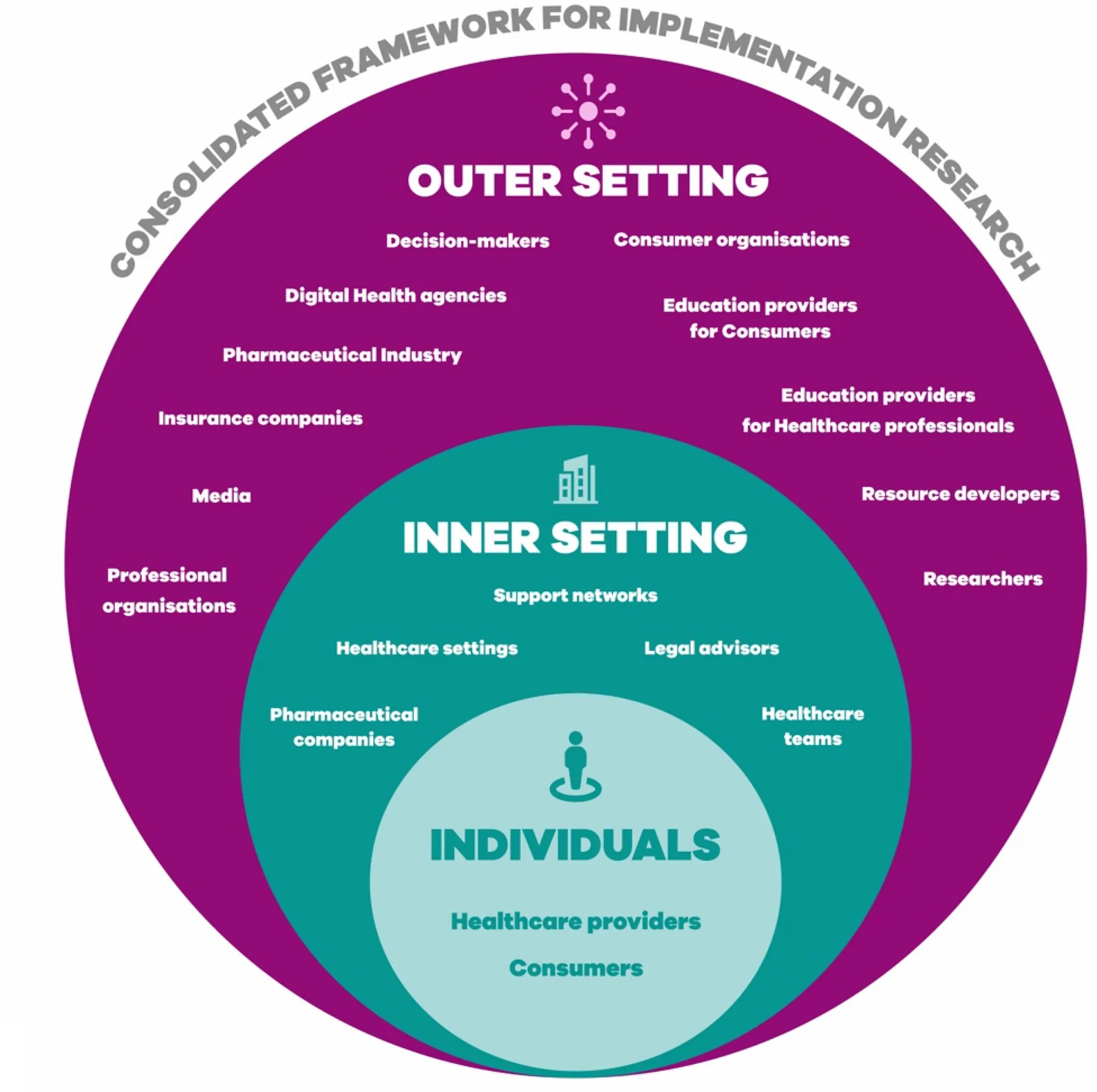
Stakeholder groups relevant to deprescribing identified from participant responses and mapped to CFIR domains. Figure (adapted from Damschroder et al. 2009) [18] illustrates the key stakeholders for deprescribing identified in this study. The Outer setting describes external factors, such as policies and incentives, influencing the Inner Setting, which reflects the internal environment determining whether change is feasible, supported, and sustainable (e.g. organisational culture). Characteristics of Individuals comprised healthcare providers directly involved in patient care and consumers who play an active role in decision-making about their medications

The boundaries between CFIR domains are often unclear [28], requiring clear delineation to support consistent interpretation. Two authors (HWQ, AP) jointly reviewed all open-text responses to identify referenced stakeholder groups and mapped them to three CFIR domains. Disagreements were resolved through discussions with other authors (KL, CEB). A concentric circle diagram was developed to visualise stakeholders’ sphere of influence as conceptualised by CFIR (Fig. 2).

### Phase II (inductive development of themes)

Inductive thematic analysis followed Braun and Clarke’s approach [29]. Responses were organised into two categories: (i) consumer-reported barriers, (ii) professional/policy stakeholder-reported barriers. Responses from participants self-identifying with dual roles were categorised according to response content. The lead author (HWQ) familiarised herself with the dataset, generated initial codes, and developed candidate themes through iterative review and discussion with all authors.

### Phase III (deductive mapping)

Barrier themes were independently mapped by two researchers (XR and HWQ) to CFIR version 1.0 constructs, as required by the CFIR-ERIC Matching Tool [18, 20]. CFIR Construct Rating Guidelines [30] were not applied, as the aim was to identify barriers rather than assess determinant strength or valence. CFIR version 1.0 was used to ensure compatibility with the Matching Tool [18]. Facilitators were also mapped but not the focus of the current reporting as the Matching Tool currently incorporates only barriers as inputs.

### Phase IV (CFIR-ERIC implementation strategies)

Barrier constructs across both stakeholder groups were combined and entered into the Matching Tool [20–22]. Strategies were ranked according to cumulative expert endorsement scores. The ten highest-ranked strategies were defined as prioritised strategies and grouped into clusters using Waltz et al.’s concept mapping framework [22], incorporating importance and feasibility ratings (Supplementary File 2, Figure S1). Facilitator constructs were triangulated with strategy clusters to provide contextual interpretation, given limited guidance on integrating facilitators into strategy selection [31].

### Research trustworthiness

Trustworthiness was guided by Lincoln and Guba’s criteria for credibility, transferability, dependability, and confirmability [32]. Credibility was supported through prolonged data engagement, iterative coding, independent mapping, and consensus discussions. Transferability was supported through detailed reporting of context, sampling, methods, findings, and illustrative quotes. Transparency was further enhanced through publication of a comprehensive report. Confirmability and dependability were maintained through an audit trail of analytical decisions, code development, and team discussions. Member checking was not undertaken to minimise respondent burden.

### Ethics approval

Ethics approval was obtained from the University of Western Australia Human Research Ethics Committee (ref: 2023/ET001118). Written informed consent was obtained electronically from all participants. Participants were asked whether they would like their response to be published in full or used for research purposes only. Direct quotes were not included in this paper where consent for full verbatim publication was not obtained.

## Results

### Characteristics of respondents

Of the 313 participants who completed the consultation survey, 248 provided responses to one or more open-ended questions on implementation barriers and enablers, which were analysed in the present study. Their characteristics are reported in Table 2. For comparison, characteristics of the 65 participants who did not respond to the optional implementation questions and the full sample of 313 participants are reported in Supplementary File 2 (Tables S2 and S3). No major differences in participant characteristics were observed between groups.

**Table 2:** Participant characteristics

Characteristics	Consumer and community* (*n* = 220)	Professional and policy stakeholder ^#^ (*n* = 16)	Combination of roles (*n* = 12)	Total (*n* = 248)
*Age groups*				
30–39	5 (2.27%)	5 (31.25%)	1 (8.33%)	11 (4.44%)
40–49	15 (6.82%)	3 (18.75%)	1 (8.33%)	19 (7.66%)
50–59	12 (5.45%)	4 (25.00%)	4 (33.33%)	20 (8.06%)
60–69	45 (20.45%)	2 (12.50%)	1 (8.33%)	48 (19.35%)
70+	143 (65.00%)	2 (12.50%)	5 (41.67%)	150 (60.48%)
*Sex*				
Female	121 (55.00%)	12 (75.00%)	8 (66.66%)	141 (56.85%)
Male	95 (43.18%)	2 (12.50%)	4 (33.33%)	101 (40.73%)
Non-binary	1 (0.45%)	0	0	1 (0.40%)
Prefer not to say	3 (1.36%)	2 (12.50%)	0	5 (2.02%)
*Population categories*				
Aboriginal and Torres Strait Islander peoples	1 (0.45%)	0	0	1 (0.40%)
LGBTQIA+ (Lesbian, Gay, Bisexual, Transgender, Queer, Intersex, and Asexual, and others)	6 (2.73%)	2 (12.50%)	0	8 (3.23%)
People from culturally and linguistically diverse communities	6 (2.73%)	2 (12.50%)	1 (8.33%)	9 (3.63%)
People living in a rural or remote area	17 (7.73%)	0	4 (33.33%)	21 (8.47%)
Prefer not to say	6 (2.73%)	4 (25.00%)	0	10 (4.03%)
None of the above	184 (83.64%)	8 (50.00%)	7 (58.33%)	199 (80.24%)

Data are presented as n (%). Some percentages do not sum to 100% due to rounding

^*^Consumers and community members were defined as those with lived or living experience, carers, family members or relatives of long-term medicine users, consumer advocates, or interested members of the public

^#^Professional and policy stakeholders included those with a work-related interest in deprescribing (i.e. healthcare professionals, health policymakers, service providers, and researchers/academics)

### Phase I: Stakeholders’ context in implementation

Figure 2 illustrates how stakeholders identified from participants’ responses were positioned within the deprescribing implementation context using the CFIR framework.

### Phase II: Themes on barriers to deprescribing

Barriers identified across both consumer and professional/policy stakeholder groups were organised into three shared themes: ‘Knowledge gaps, beliefs, or limited readiness’, ‘Communication, hierarchy, or social influences limiting deprescribing’, and ‘System-level structures misaligned with deprescribing practices’. Consumers additionally identified barriers related to a lack of person-centred and adaptable approaches to deprescribing, whereas professional/policy stakeholders described barriers related to medicine (or drug) formulations and guideline characteristics (Table 3).

**Table 3:** Barrier themes mapped to CFIR domains and constructs

Barrier themes	CFIR domains	CFIR constructs	Barrier subtheme (quote references)
Lack of person-centred and adaptable approach to deprescribing	Intervention characteristics	Evidence strength & quality; Adaptability; Trialability	Guideline recommendations and tapering approaches do not tailor to individual circumstances [C1, C2, C3]
	Outer setting	Patient needs & resources	Person-centred care overlooked in favour of generalised evidence and standardised study findings [C17]
Knowledge gaps, beliefs, and limited readiness to deprescribe	Intervention characteristics	Relative advantage	Risks of deprescribing outweigh the benefits [C6]
Unsuitable medicine (or drug) formulation and guideline format affecting guideline implementation	Intervention characteristics	Adaptability; Complexity; Design quality and packaging	Limited adaptability of tapering approaches [P1] Complexity affecting guideline usability [P2.1] Limited compatibility of guideline format with practice contexts [P3]
System-level structures misaligned with deprescribing practices	Intervention characteristics	Cost	Economic barriers affecting deprescribing or guideline implementation [C22, P5]
	Outer setting	External policy & incentives	Insufficient legal protection and regulatory oversight, and competing system or commercial priorities influencing prescribing decisions [C18, C19, C20, P2.2]
	Inner setting	Structural characteristics; Tension for change; Compatibility; Organisational incentives & rewards; Available resources; Access to knowledge & information	Structural, digital, and workflow constraints limiting integration of deprescribing into routine practice [P4.4, P7, C24] Unsupportive practice climate for implementation [P6, C25] Pharmaceutical influence/incentive on prescribing [C21] Insufficient resources or access to information or knowledge to support deprescribing [C23.1, C23.2, C26, P2.3, P4.5]
Communication, hierarchy, or social influences limiting deprescribing	Inner setting	Networks & communications; Culture; Implementation climate; Leadership engagement	Communication gaps [C11, C15] Power imbalances [C12] Entrenched prescribing norms [C13, P4.2] Social influences [C16, P4.3] Low implementation climate limiting shared decision-making and willingness to deprescribe [C14]
	Characteristics of individuals	Knowledge & beliefs about the intervention; Self-efficacy; Individual stage of change	Limited knowledge on deprescribing and existing beliefs about deprescribing and medication use contributing to low self-efficacy and willingness among consumers and clinicians to engage in deprescribing [C4, C5, C7, C8, C9, C10, P4.1]

***C*** refers to quotes from consumers and community members; ***P*** refers to quotes from professional and policy stakeholders. Illustrative quotes are presented in the narrative text rather than reproduced here. Where a participant contributed more than one quote, quotes are numbered sequentially (e.g., C23.1, C23.2)

### Consumers’ perspectives

#### Theme 1: Lack of person-centred and adaptable approach to deprescribing

Consumers emphasised the importance of individualised approaches to deprescribing. Some consumers expressed concern that guideline recommendations may not adequately account for individual risks and experiences, particularly where medication changes could have unintended consequences:

“Forced deprescribing in older adults can be deadly. These recommendations overlook long-term harm and risk injuring the very population they aim to help.” (Consumer participant 1, C1)

Consumers also described the need for adaptable deprescribing approaches, including clearer communication of risks before starting a medicine and opportunities to tailor tapering approaches over time and questioned whether dose reduction approaches could be adapted to individual needs:

“I would have loved to have had a warning because I never even knew [of] the dangers [of benzodiazepines] until it was too late.” (C2)

“… Try get off [too] quick or just cut in half or scrap/bite off or reduce 1 mg a week or 2.” (C3)

#### Theme 2: Consumer knowledge gaps, beliefs, and limited readiness to deprescribe

Consumers identified limited knowledge about deprescribing and uncertainty about medicine-related decisions as barriers. Some participants described insufficient awareness of deprescribing principles:

“Not having enough knowledge on the subject.” (C4)

Others described established beliefs favouring continued medicine use, particularly when medicines had been associated with symptom control after prolonged adjustment periods.

“[A] pill for every ill [perspective].” (C5)

“It took a long time… to find a treatment regime that… didn't sicken me.” (C6)

Limited confidence in initiating deprescribing discussions with healthcare providers was also reported, with some consumers perceiving themselves as lacking authority to question clinical decisions.

“I would not be comfortable discussing the guidelines with my cardiologist.” (C7)

Consumers also perceived clinician reluctance and variable readiness to deprescribe as influencing opportunities for discussion.

“[Healthcare professionals’] reluctance to deprescribe.” (C8)

“Conflict between the dyed in the wool people and the innovators.” (C9)

“My doctor is old and probably unwilling or too busy to read up [on] the data [presented in the guideline].” (C10)

#### Theme 3: Communication, hierarchy, or social influences limiting deprescribing

Consumers described communication barriers that restricted opportunities for shared decision-making, including inadequate explanations, one-way communication, and language or health literacy challenges:

“Language barriers for people from CALD [culturally and linguistically diverse backgrounds] etc.” (C11)

Participants also perceived power imbalances within healthcare relationships, where established prescribing norms and professional authority could constrain deprescribing discussions.

“Psychiatrists and their overall power.” (C12)

“Entrenched views about blood pressure and cholesterol amongst medical professionals.” (C13)

Some consumers perceived that healthcare professionals were not always receptive to deprescribing discussions, creating uncertainty about whether concerns could be raised.

“He [their healthcare professional] might not think [discussing deprescribing is] appropriate.” (C14)

Fragmented communication and interprofessional relationships were also perceived to limit deprescribing.

“Lack of communication between the hospital and GP [general practitioners].” (C15)

“Where the prescription was recommended by the GP, specialist[s] are reluctant to overturn the GP advice.” (C16)

#### Theme 4: System-level structures misaligned with deprescribing practices

Consumers identified system-level barriers related to existing healthcare models, policy, funding, workforce capacity, and information availability. Participants perceived that the current healthcare model did not support person-centred decision-making:

“The healthcare system lacks holistic person-centred care … and that the individual is often disregarded in favour of ‘studies’ that might suggest otherwise, in terms of the patient's experience.” (C17)

Structural concerns limiting deprescribing decision-making included fear of legal consequences, variable monitoring of care standards, and perceived commercial influences.

“Fear of legal ramifications.” (C18)

“… [There] are changes happening to [aged] care, but nursing staff are not being monitored by the ACSQHC [Australian Commission on Safety and Quality in Health Care] or ACQSC [Aged Care Quality and Safety Commission]. Standards are lacking due to a range of issues.” (C19)

Some consumers expressed concerns that commercial interests may influence prescribing practices:

“… if the health professional has a vested interest in the pharmaceutical industry.” (C20)

“Doctors prescribe medications that are promoted by sales [representatives] from pharmacies.” (C21)

Financial and practical barriers, including consultation costs, limited healthcare professional availability, and competing demands on healthcare professionals’ time, were perceived to restrict deprescribing discussions:

“Most GPs don't bulk bill for appointments, which makes it very expensive to access their services.” (C22)

“I feel dubious about anything that is required of them that is time consuming.” (C23.1)

“The wait time between requesting a conversation with [the] in-house GP and fulfilling the request is frustrating.” (C24)

Consumers further highlighted a lack of focus on deprescribing in healthcare professional education and training, alongside insufficient access to information and resources to support deprescribing, for example:

“It's not part of their educational business model.” (C25)

“… [clinicians may not be] fully acquainted with my other conditions and role of the specific medications.” (C23.2)

“Lack of resources to help them [their healthcare professionals].” (C26)

### Perspectives from professional and policy stakeholders

#### Theme 1: Unsuitable medicine (or drug) formulation and guideline format affecting guideline implementation

Professional and policy stakeholders identified practical implementation barriers related to medicine formulations, guideline applicability, and usability. Challenges were particularly noted for deprescribing processes requiring gradual dose reductions, with limited access to suitable formulation options perceived as a barrier. A tapering coach noted:

“Do not have access to compounding pharmacies to obtain special capsules.” (Professional/policy stakeholder participant 1, P1)

Beyond formulation issues, stakeholders also described challenges associated with applying a guideline intended for use across diverse healthcare professions and settings, suggesting that broad applicability may reduce ease of uptake. A health services researcher reflected:

“The intentional applicability of the guideline to assist healthcare providers across multiple professions and settings may be a barrier to uptake.” (P2.1)

Differences in guideline format and accessibility were also perceived to influence usability, particularly across digital, print, and time-constrained clinical environments. For instance, a medical practitioner expressed:

“ ... might only work if accessed via a screen.” (P3)

#### Theme 2: Clinicians’ knowledge gaps, beliefs, and limited readiness to deprescribe

Professional and policy stakeholders identified gaps in clinicians’ knowledge, confidence, and beliefs about deprescribing that may limit their readiness to deprescribe. A health policy professional noted:

“Prescribers may be unfamiliar with common drug-drug interactions and prescribing cascades … concerns regarding the potential negative consequences arising from ceasing medications.” (P4.1)

#### Theme 3: Communication, hierarchy, or social influences limiting deprescribing

The same health policy professional perceived entrenched hierarchical relationships, prescribing norms, and social influences as barriers to deprescribing. Entrenched beliefs about prescribing and expectations of clinician authority were perceived to reinforce continuation of medicines. Resistance from family members and carers was also identified as a potential barrier to deprescribing discussions and decisions.

“Doctor knows best… white coat syndrome… [and] cultural norms that encourage medication prescribing rather than deprescribing.” (P4.2)

“Resistance from family members or carers.” (P4.3)

#### Theme 4: System-level structures misaligned with deprescribing practices

Professional and policy stakeholders identified system-level barriers related to funding models, workforce capacity, competing priorities, workflows, education, and access to information. Financial and time constraints were perceived to limit clinicians’ capacity to engage in deprescribing activities, particularly where these activities were not explicitly reimbursed or supported. For example, the health services researcher and medical practitioners explained:

“… generic challenges within the healthcare system such as limited capacity, competing priorities.” (P2.2)

“[GPs were expected to] self-fund all [their] CPD [Continuing Professional Development] which represents a drain on … time and finances.” (P5)

“[Limited] time in consultations.” (P6)

Organisational processes and digital systems were perceived as contributing to reinforce continuation rather than deprescribing, as noted by health services and policy professionals:

“… ease of continuing prescribing in digital systems through alert fatigue,” (P4.4)

“Workflow integration limitations (i.e. suboptimal change management practices).” (P7)

Stakeholders further identified gaps in education, training, supportive tools, and access to complete patient information as barriers to informed deprescribing decisions, as noted by health services and policy professionals:

“Lack of training among primary healthcare practitioners to adequately address polypharmacy” [P4.5],

“… if the health professional does not have access to robust evidence regarding their patients’ medical history, this will significantly impact their ability to implement the guidelines as they will not have the required evidence to make informed decisions.” (P2.3)

### Phase III: Stakeholder-identified barriers mapped to CFIR

Barriers reported by consumers and professional and policy stakeholders were mapped to 18 and 14 CFIR constructs (Table 3). Across stakeholder groups, barriers mapped predominantly to the CFIR Inner Setting, with additional barriers identified across Intervention characteristics, Outer setting, and Characteristics of individuals. Intervention-related barriers reflected concerns regarding the evidence strength and quality, adaptability, trialability, relative advantage, complexity, and design quality and packaging of the guideline, including their compatibility with individual circumstances and clinical practice contexts. Outer setting barriers included patient needs and resources, as well as external policies, incentives, and cost considerations. Inner setting barriers encompassed structural characteristics, tension for change, compatibility with existing workflows, organisational incentives and rewards, available resources, access to knowledge and information, networks and communication, culture, implementation climate, and leadership engagement. Barriers mapped to Characteristics of individuals included knowledge and beliefs about the intervention, self-efficacy, and readiness to engage in deprescribing.

### Phase IV: Expert-endorsed ERIC implementation strategies generated using CFIR barriers

Combining the CFIR constructs identified across both stakeholder groups and inputting them into the CFIR-ERIC Matching Tool resulted in a list of implementation strategies based on cumulative expert endorsement (Table 4). Expert endorsement reflects the extent to which CFIR implementation experts recommended each ERIC strategy as relevant for addressing the identified CFIR barriers. The prioritised strategies from the Matching Tool were broadly consistent with facilitators identified by stakeholders, which commonly mapped to the CFIR Inner setting. The facilitator themes most commonly aligned with strategy clusters focused on developing stakeholder interrelationships, engaging consumers, and training and educating stakeholders (Supplementary File 2, Tables S4 and S5).

**Table 4:** Resulting CFIR-ERIC strategies, organised by clusters

Strategy cluster^†^	Ranked CFIR-ERIC strategies (cumulative percentage of expert endorsements, %)	Top three targeted barrier constructs (expert endorsement, %)	Relative importance ratings^†^	Relative feasibility ratings^†^	Go-zone quadrant^†^
Develop stakeholder interrelationships	#1 Identify and prepare champions (614%)	Culture (52%); Tension for change (48%); Relative advantage (45%)	4.20	3.77	I
	#4 Conduct local consensus discussions (430%)	Tension for change (43%); Compatibility (41%); Evidence strength & quality (41%)	3.63	4.07	I
	#6 Capture and share local knowledge (418%)	Adaptability (35%); Access to knowledge & information (31%); Complexity (27%)	3.63	3.87	I
	#9 Inform local opinion leaders (379%)	Tension for change (39%); Evidence strength & quality (38%); Knowledge & beliefs about the intervention (28%)	3.90	4.03	I
Use evaluative and iterative strategies	#2 Assess for readiness and identify barriers and facilitators (493%)	Implementation climate (52%); Culture (41%); Structural characteristics (36%)	4.60	4.57	I
	#10 Conduct local need assessment (367%)	Patient needs & resources (57%); Tension for change (43%); Adaptability (35%)	4.27	4.33	I
Adapt and tailor to context	#3 Promote adaptability (447%)	Adaptability (73%); Design quality & packaging (48%); Compatibility (45%)	3.90	3.57	I
Train and educate stakeholders	#5 Conduct educational meetings (424%)	Access to knowledge & information (79%); Knowledge & beliefs about the intervention (56%); Evidence strength & quality (47%)	3.27	4.50	I
	#8 Create a learning collaborative (388%)	Access to knowledge & information (45%); Networks & Communications (35%); Complexity (33%)	3.10	3.43	II
Utilise financial strategies	#7 Alter incentive/allowance structures (413%)	Organisational incentives & rewards (71%); Implementation climate (44%); Cost (44%)	3.17	2.23	III

CFIR, Consolidated Framework for Implementation Research; ERIC, Expert Recommendations for Implementing Change

^†^CFIR-ERIC strategies presented are based on barriers identified and mapped using the CFIR-ERIC Matching Tool in Phase III [1–3]. Cumulative percentage endorsements exceed 100% because expert endorsement ratings for the same ERIC strategy are aggregated across multiple CFIR barrier constructs. Strategies were grouped into clusters according to Waltz et al. concept mapping framework [3]. Importance was rated on a scale from 1 (relatively unimportant) to 5 (extremely important), and feasibility was rated from 1 (not at all feasible) to 5 (extremely feasible). Go-zone quadrants combine these ratings, with quadrant I represents high importance/high feasibility, quadrant II low importance/high feasibility, quadrant III low importance/low feasibility, and quadrant IV high importance/low feasibility

## Discussion

This study identified that barriers to deprescribing guideline implementation were predominantly situated within the CFIR Inner Setting domain, highlighting that successful implementation depends not only on evidence-based recommendations but also on organisational conditions. Challenges related to stakeholder relationships, communication, culture, implementation climate, workflow, leadership, resources, and information access suggest that deprescribing represents a complex practice change requiring alignment across consumers, clinicians, and healthcare organisations. Accordingly, ERIC strategies prioritise stakeholder interrelationships, local adaptation, and iterative approaches to support implementation.

The barriers described by stakeholders were consistent with previous literature. Consumers reported concerns regarding deprescribing appropriateness, fear of stopping medicines, healthcare system complexity, and limited healthcare professional support [33–35]. Professional stakeholders similarly highlighted limited confidence, insufficient communication, and practice environments that do not support deprescribing [36, 37]. Implementation research in deprescribing and polypharmacy management further reinforces the importance of addressing Inner setting barriers, including relational, organisational, and contextual influences on sustainable practice change [24, 38].

Although ‘Identify and prepare champions’ was the highest-ranked strategy, consistent with evidence highlighting local leadership in practice change [39, 40], the diverse CFIR-ERIC strategy clusters indicate that deprescribing requires a multifaceted approach. Champions may facilitate engagement, address resistance, and embed new practices within organisational culture [20]. However, sustaining their impact requires complementary strategies. Other highly endorsed strategies, including assessing readiness, identifying barriers and facilitators, promoting adaptability, conducting local consensus discussions, providing educational meetings, and sharing local knowledge, may strengthen implementation capacity and alignment with local needs. This aligns with a recent champion-led deprescribing study in people with dementia demonstrating the need for multifaceted approaches to support champions in overcoming contextual barriers [41]. As determinants may differ between settings [42] and shift over time [43], ongoing assessment and local adaptation are required.

Although the CFIR-ERIC Matching Tool has increasingly guided implementation planning in primary care since 2019, evidence evaluating whether theory-informed strategies improve implementation or clinical outcomes remains limited [23]. One study found that framework‑recommended strategies were more strongly associated with improved cirrhosis care than actual strategies used in practice [43]. Future research should prospectively evaluate the feasibility, acceptability, fidelity, sustainability, and effectiveness of these strategies in real-world settings. Frameworks such as RE-AIM could guide evaluation of reach, effectiveness, adoption, implementation, and maintenance [44]. Generating practice-based evidence could inform implementation, scale-up, and sustainable integration [10].

The study’s main strength is the use of an implementation science framework linking stakeholder-identified barriers with theory-informed strategies [45]. The inclusion of substantial consumer input provides important insights for person-centred deprescribing approaches. However, limitations should be acknowledged. The public consultation enabled broad stakeholder engagement but may have introduced selection bias, as respondents were self-selected and may have held stronger views on deprescribing. Minority groups were underrepresented, and thematic saturation was not assessed because the aim was to maximise inclusion of consultation feedback. As consumers were consulted as recipients rather than implementers of deprescribing, CFIR construct-specific questions [30] were not used. Instead, open-ended responses enabled participant-driven identification of barriers followed by inductive and deductive mapping. While this approach supported stakeholder-driven insights, it may have captured less contextual detail than construct-specific questioning. The questionnaire was not formally validated or pilot tested, potentially limiting opportunities for refinement. Finally, CFIR-ERIC strategy selection relies on expert endorsement and judgement, introducing potential subjectivity, and expert ratings vary regarding which strategies best address specific barriers [21]. The tool also does not incorporate facilitators, potentially limiting alignment between determinants and recommended strategies. Given increasing recognition of strength-based approaches incorporating both barriers and facilitators [31], the resulting strategies should be interpreted as theory-informed priorities rather than definitive recommendations.

## Conclusion

Findings demonstrate that deprescribing barriers are frequently located within the CFIR Inner Setting domain across stakeholder groups. CFIR-ERIC strategies offer theory-informed implementation priorities, with emphasis on stakeholder engagement and local capacity building. Future research should focus on adapting these strategies to different healthcare settings and evaluating their impact on implementation, healthcare delivery, and person-centred care.

## Supplementary Information

Below is the link to the electronic supplementary material.

Supplementary file1 (PDF 3712 KB)

Supplementary file2 (PDF 315 KB)

Supplementary file3 (PDF 1299 KB)

## Data Availability

Further data that support this study may be available upon reasonable request from the corresponding author. No datasets were generated or analysed during the current study.
