# Supplementary material for: A qualitative analysis to identify implementation strategies for a deprescribing clinical practice guideline using the CFIR-ERIC Matching Tool: perspectives from consumers and professional stakeholders
Source: Int J Clin Pharm. 2026 Sep 3;48(6):2403–17. doi: 10.1007/s11096-026-02218-9 (PMC13731450; doi:10.1007/s11096-026-02218-9)
Supplement: Supplementary file 1 [file 11096_2026_2218_MOESM1_ESM.pdf]

Supplementary Material

Manuscript title: A qualitative analysis to identify implementation strategies for a deprescribing clinical practice guideline using the CFIR-ERIC Matching Tool: perspectives from older people and professional stakeholders

This supplementary file provides screenshots of the webpage used to disseminate the public consultation, which was open from 24 April to 31 May 2025. The working draft guideline and associated supporting reports were made available for stakeholder review and comment via this dedicated consultation webpage: *https://deprescribing.com/about/documents-for-public-consultation.html*

The full public consultation report, including a summary of feedback received and responses to submissions, is available at: *https://deprescribing.com*

dRx

Listen to this podcast episode to learn more about the guideline.

PUBLIC

CONSULTATION

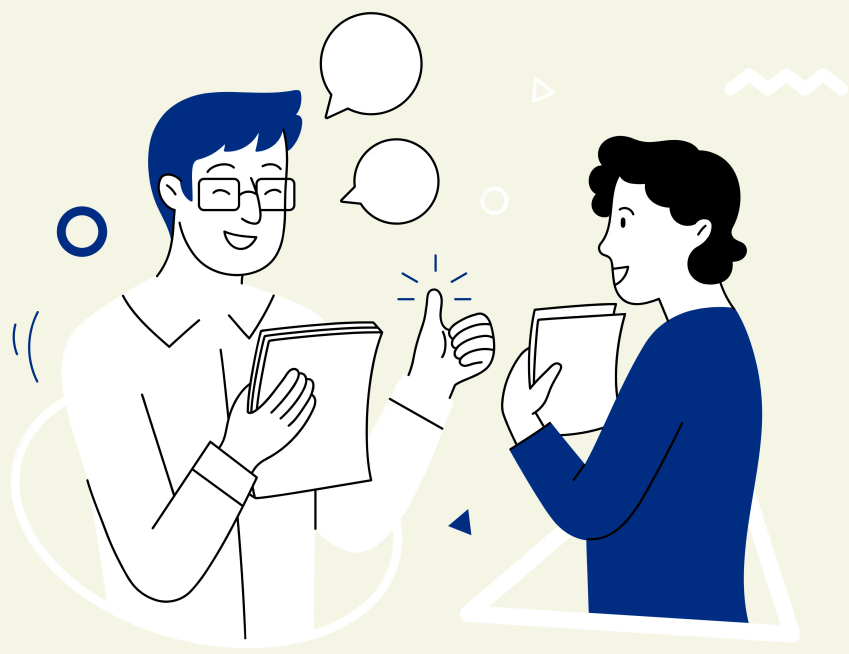

DRAFT Clinical Practice Guidelines for Deprescribing in Older People

Thank you for your interest in the review of the DRAFT guidelines for deprescribing in older people.

The call for public submissions is now closed.

We appreciate all input received.

Feedback is currently being reviewed and will help shape the final version of the guidelines.

All submitted responses will be made publicly available on this website, where consent has been given to publish the response.

Closed 31 May 2025

Opened 24 April 2025

Contact

deprescribing@uwa.edu.au

Who can make a submission?

Anyone with an interest in deprescribing is invited to make a submission. This includes:

- Consumers/carers/family members with lived or living experience
- Individuals with an interest

Listen to this podcast episode to learn more about the guideline.

Submissions can be made by individuals or on behalf of an organisation.

How do I make a submission?

The call for public submissions is now closed.

What questions will be asked?

The submission form includes both general and specific questions about the DRAFT Guidelines. You are not required to respond to every question – please focus on areas that are most relevant or important to you.

You may wish to consider:

1. Is the guideline clear and easy to follow? If not, which sections need improvement?
2. Are there any terms or concepts that require further clarification?
3. Does the guideline address key concerns relevant to your role or experience?
4. Is there sufficient detail to support decision-making for deprescribing? What additional information would be helpful?
5. How do you envision this guideline being used in practice?

Why your views matter

Your input will play a crucial role in refining the final version of the guidelines. All feedback will be carefully reviewed and considered. A report of public consultation will be published alongside the final guideline for transparency. Your responses will remain confidential, and your identity will not be linked to any of the feedback you provide unless you provide consent.

Ethics approval

Ethics approval has been granted by the University of Western Australia Human Research Ethics Committee (Ref: 2023/ET001118).

Contacts

If you experience any issues accessing any documents, please email us and we will provide them in an alternate format.

📩 General enquiries: deprescribing@uwa.edu.au

Listen to this podcast episode to learn more about the guideline.

The following draft documents are available for download:

DRAFT Deprescribing Guidelines

Recommendations for deprescribing in older people

Download

For online website versions, click the links below:

- Consumer Preface
- Plain Language Summary
- Executive Summary
- Introduction
- Polypharmacy / Multiple Drug Classes
- Individual Drug Classes

DRAFT Technical Report

Describes the methodology used in the development of the deprescribing guidelines, including evidence review and recommendation formulation process.

Download

DRAFT Administrative Report

Documents the non-technical aspects of guideline development, including information on the governance structure, funding sources, development timelines, stakeholder involvement, and the processes used to declare and manage conflicts of interest.

Download

DRAFT Dissemination and Implementation Plan

Outlines proposed strategies for disseminating and implementing the guideline in practice.

Download

Listen to this podcast episode to learn more about the guideline.

Quick Links

Our People

Funding

Other Publications

Contact Us

deprescribing@uwa.edu.au

Send us Feedback

We acknowledge the Traditional Owners of Country throughout Australia and acknowledge their continuing connection to land, waters and community. We pay our respects to the people, the cultures and the Elders past and present.

The information and recommendations presented in this guideline represent the view of the guideline development group, based on careful consideration of the available evidence. It is not mandatory to apply the recommendations, and the guideline does not replace clinical judgment or the responsibility to make decisions tailored to the individual's circumstances, in consultation with the person, their family, and/or carers.

Copyright © 2026 Western Australia Centre for Health and Ageing and Centre for Optimisation of Medicines, The University of Western Australia
