# Supplementary material for: A qualitative analysis to identify implementation strategies for a deprescribing clinical practice guideline using the CFIR-ERIC Matching Tool: perspectives from consumers and professional stakeholders
Source: Int J Clin Pharm. 2026 Sep 3;48(6):2403–17. doi: 10.1007/s11096-026-02218-9 (PMC13731450; doi:10.1007/s11096-026-02218-9)
Supplement: Supplementary file 2 [file 11096_2026_2218_MOESM2_ESM.pdf]

Supplementary File 2

Table S1. Standards for Reporting Qualitative Research (SRQR)

	**Section/ Page number**
**Title and abstract**	
**Title -** Concise description of the nature and topic of the study Identifying the study as qualitative or indicating the approach (e.g., ethnography, grounded theory) or data collection methods (e.g., interview, focus group) is recommended	Title
**Abstract -** Summary of key elements of the study using the abstract format of the intended publication; typically includes background, purpose, methods, results, and conclusions	Abstract
**Introduction**	
**Problem formulation -** Description and significance of the problem/phenomenon studied; review of relevant theory and empirical work; problem statement	Introduction
**Purpose or research questio**n **-** Purpose of the study and specific objectives or questions	Introduction
**Methods**	
**Qualitative approach and research paradigm -** Qualitative approach (e.g., ethnography, grounded theory, case study, phenomenology, narrative research) and guiding theory if appropriate; identifying the research paradigm (e.g., postpositivist, constructivist/ interpretivist) is also recommended; rationale**	Study design and context
**Researcher characteristics and reflexivity -** Researchers' characteristics that may influence the research, including personal attributes, qualifications/experience, relationship with participants, assumptions, and/or presuppositions; potential or actual interaction between researchers' characteristics and the research questions, approach, methods, results, and/or transferability	Researcher characteristics
**Context -** Setting/site and salient contextual factors; rationale**	Study design and context
**Sampling strategy -** How and why research participants, documents, or events were selected; criteria for deciding when no further sampling was necessary (e.g., sampling saturation); rationale**	Study design and context
**Ethical issues pertaining to human subjects -** Documentation of approval by an appropriate ethics review board and participant consent, or explanation for lack thereof; other confidentiality and data security issues	Ethics approval
**Data collection methods -** Types of data collected; details of data collection procedures including (as appropriate) start and stop dates of data collection and analysis, iterative process, triangulation of sources/methods, and modification of procedures in response to evolving study findings; rationale**	Study design and context, Questionnaire

	development and administration
**Data collection instruments and technologies** - Description of instruments (e.g., interview guides, questionnaires) and devices (e.g., audio recorders) used for data collection; if/how the instrument(s) changed over the course of the study	Questionnaire development and administration
**Units of study** - Number and relevant characteristics of participants, documents, or events included in the study; level of participation (could be reported in results)	Questionnaire development and administration
**Data processing** - Methods for processing data prior to and during analysis, including transcription, data entry, data management and security, verification of data integrity, data coding, and anonymization/de-identification of excerpts	Data analysis
**Data analysis** - Process by which inferences, themes, etc., were identified and developed, including the researchers involved in data analysis; usually references a specific paradigm or approach; rationale**	Data analysis
**Techniques to enhance trustworthiness** - Techniques to enhance trustworthiness and credibility of data analysis (e.g., member checking, audit trail, triangulation); rationale**	Research trustworthiness
**Results/findings**	
**Synthesis and interpretation** - Main findings (e.g., interpretations, inferences, and themes); might include development of a theory or model, or integration with prior research or theory	Results
**Links to empirical data** - Evidence (e.g., quotes, field notes, text excerpts, photographs) to substantiate analytic findings	Results and Table 3
**Discussion**	
**Integration with prior work, implications, transferability, and contribution(s) to the field** - Short summary of main findings; explanation of how findings and conclusions connect to, support, elaborate on, or challenge conclusions of earlier scholarship; discussion of scope of application/generalizability; identification of unique contribution(s) to scholarship in a discipline or field	Discussions
**Limitations** - Trustworthiness and limitations of findings	Discussions
**Other**	
**Conflicts of interest** - Potential sources of influence or perceived influence on study conduct and conclusions; how these were managed	Competing interests, Pg 13
**Funding** - Sources of funding and other support; role of funders in data collection, interpretation, and reporting	Funding, Pg 13

Reference: O'Brien BC, Harris IB, Beckman TJ, Reed DA, Cook DA. Standards for reporting qualitative research: a synthesis of recommendations. Acad Med. 2014 Sep;89(9):1245-51. https://doi.org/10.1097/ACM.0000000000000388

Figure S1. Go-zone quadrants based on the mean importance and feasibility ratings

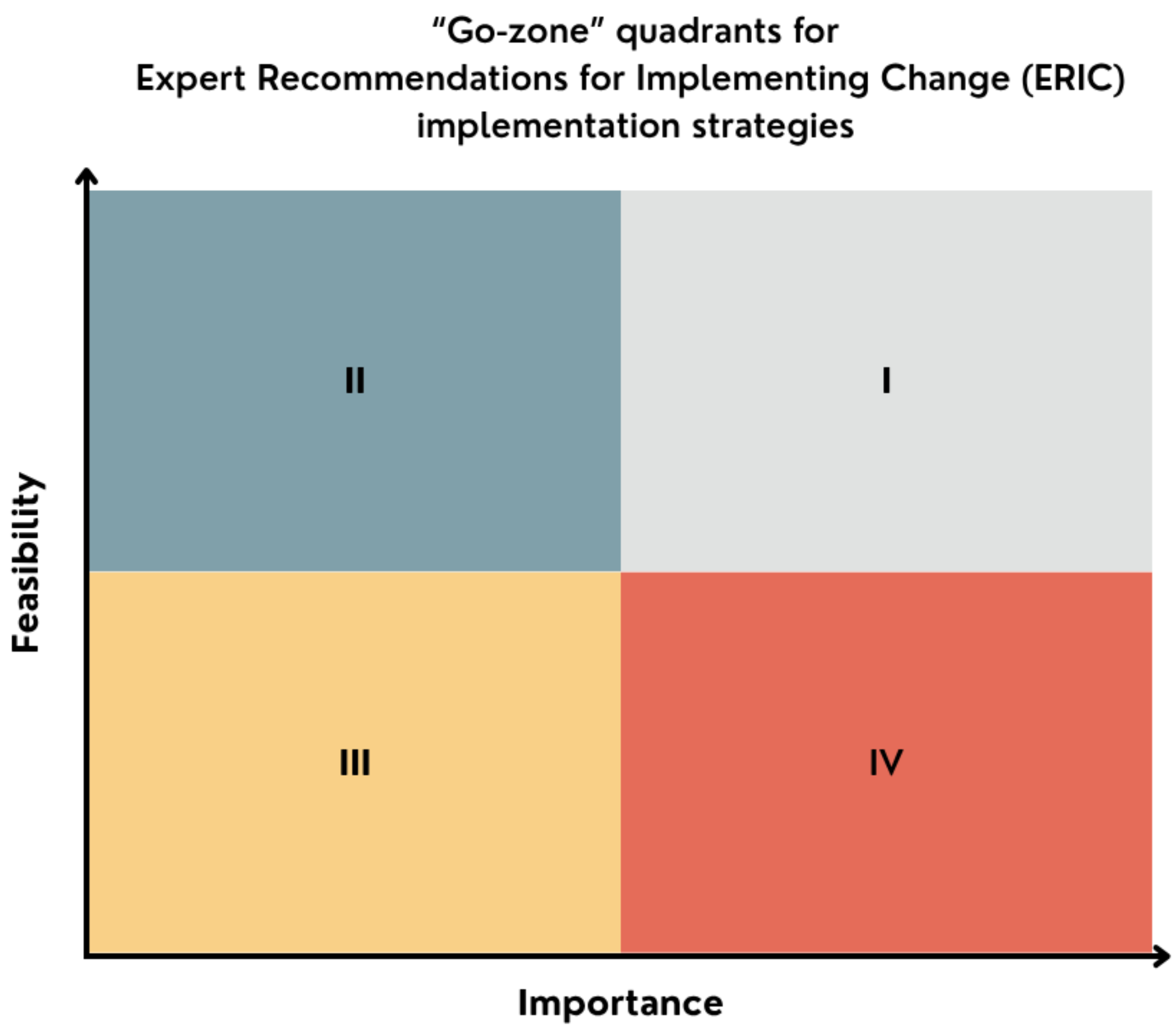

Figure adapted from Waltz et al. 2015. "Go-zone plot" showing all implementation strategies based on expert ratings of importance and feasibility. The plot is divided into four quadrants using the overall mean scores for both importance and feasibility as cut-off points. These cut-off values are not shown in the figure for simplicity. Quadrant I represents strategies rated as both high importance and high feasibility; Quadrant II represents lower importance but high feasibility and Quadrant III represents low importance and low feasibility; and Quadrant IV represents high importance but low feasibility. The cut-off values for the scale are omitted from the figure for simplicity.

Reference: Waltz TJ, Powell BJ, Matthieu MM, Damschroder LJ, Chinman MJ, Smith JL, et al. Use of concept mapping to characterize relationships among implementation strategies and assess their feasibility and importance: results from the Expert Recommendations for Implementing Change (ERIC) study. Implement Sci. 2015;10:109. https://doi.org/10.1186/s13012-015-0295-0

Table S2. Characteristics of the participants who did not respond to the optional implementation questions (*n*=65) and those who did (*n*=248)

**Characteristics**	**Non-responders (*n*=65)**			**Responders (*n*=248)**		
	**Consumer and community* (*n*=56)**	**Professional and policy stakeholder[#] (*n*=9)**	**Total (*n*=65)**	**Consumer and community* (*n*=220)**	**Professional and policy stakeholder[#] (*n*=28)**	**Total (*n*=248)**
Age groups						
20-29	1 (1.79%)	2 (22.22%)	3 (4.62%)	0	0	0
30-39	2 (3.57%)	0	2 (3.08%)	5 (2.27%)	6 (21.43%)	11 (4.44%)
40-49	1 (1.79%)	1 (1.79%)	2 (3.08%)	15 (6.82%)	4 (14.29%)	19 (7.66%)
50-59	0	2 (3.57%)	2 (3.08%)	12 (5.45%)	8 (28.57%)	20 (8.06%)
60-69	6 (10.71%)	4 (44.44%)	10 (15.38%)	45 (20.45%)	3 (10.71%)	48 (19.35%)
70+	46 (82.14%)	0	46 (70.77%)	143 (65.00%)	7 (25%)	150 (60.48%)
Sex						
Female	11 (19.64%)	8 (88.89%)	19 (29.23%)	121 (55.00%)	20 (71.43%)	141 (56.85%)
Male	45 (80.36%)	1 (1.79%)	46 (70.77%)	95 (43.18%)	6 (21.43%)	101 (40.73%)
Non-binary	0	0	0	1 (0.45%)	0	1 (0.40%)
Prefer not to say	0	0	0	3 (1.36%)	2 (7.14%)	5 (2.02%)
Population categories						
Aboriginal and Torres Strait Islander peoples	0	0	0	1 (0.45%)	0	1 (0.40%)
LGBTQIA+ (Lesbian, Gay, Bisexual, Transgender, Queer, Intersex, and Asexual, and others)	1 (1.79%)	0	1 (1.54%)	6 (2.73%)	2 (7.14%)	8 (3.23%)
People from culturally and linguistically diverse communities	2 (3.57%)	0	2 (3.08%)	6 (2.73%)	3 (10.71%)	9 (3.63%)
People living in a rural or remote area	5 (8.93%)	0	5 (7.69%)	17 (7.73%)	4 (14.29%)	21 (8.47%)
Prefer not to say	1 (1.79%)	0	1 (1.54%)	6 (2.73%)	4 (14.29%)	10 (4.03%)
None of the above	47 (83.93%)	9 (100%)	56 (86.15%)	184 (83.64%)	15 (53.57%)	199 (80.24%)

Data are presented as n (%). *Some percentages do not sum to 100% due to rounding.*

* Consumers and community members were defined as those with lived or living experience, carers, family members or relatives of long-term medicine users, consumer advocates, or interested members of the public.
# Professional and policy stakeholders were defined as those with a work-related interest in deprescribing (i.e. healthcare professionals, health policymakers, service providers, and researchers/academics).

Table S3. Characteristics of all participants in the public consultation (*n*=313)

**Characteristics**	**Consumer and community* (*n*=276)**	**Professional and policy stakeholder# (*n*=37)**	**Total (*n*=313)**
Age groups			
20-29	1 (0.36%)	2 (5.41%)	3 (0.96%)
30-39	7 (2.54%)	6 (1.62%)	13 (4.15%)
40-49	16 (5.80%)	5 (13.51%)	21 (6.71%)
50-59	12 (4.35%)	10 (27.03%)	22 (7.03%)
60-69	51 (18.48%)	7 (18.92%)	58 (18.53%)
70+	189 (68.48%)	7 (18.92%)	196 (62.62%)
Sex			
Female	132 (47.83%)	28 (75.68%)	160 (51.12%)
Male	140 (50.72%)	7 (18.92%)	147 (46.96%)
Non-binary	1 (0.36%)	0	1 (0.32%)
Prefer not to say	3 (1.09%)	2 (5.41%)	5 (1.60%)
Population categories			
Aboriginal and Torres Strait Islander peoples	1 (0.36%)	0	1 (0.32%)
LGBTQIA+ (Lesbian, Gay, Bisexual, Transgender, Queer, Intersex, and Asexual, and others)	7 (2.54%)	2 (5.41%)	9 (2.88%)
People from culturally and linguistically diverse communities	8 (2.90%)	3 (8.11%)	11 (3.51%)
People living in a rural or remote area	22 (7.97%)	4 (10.81%)	26 (8.31%)
Prefer not to say	7 (2.54%)	4 (10.81%)	11 (3.51%)
None of the above	231 (83.70%)	24 (64.86%)	255 (81.47%)

Data are presented as n (%). *Some percentages do not sum to 100% due to rounding.*
* Consumers and community members were defined as those with lived or living experience, carers, family members or relatives of long-term medicine users, consumer advocates, or interested members of the public.
# Professional and policy stakeholders were defined as those with a work-related interest in deprescribing (i.e. healthcare professionals, health policymakers, service providers, and researchers/academics).

Table S4. Stakeholders' perceived facilitators to deprescribing with supporting quotes, categorised based on the Consolidated Framework for Implementation Research (CFIR)

CFIR domains	CFIR constructs	Consumers and community members		Professional and policy stakeholders	
		Descriptions	Illustrative quotes	Descriptions	Illustrative quotes
**Intervention characteristics**	Evidence Strength & Quality	Evidence-based resources from trusted or reputable source	*"Results from an accredited source"*	Evidence-based and current guidelines	*"The guidelines are evidence-based and whilst there is a lot of information for each recommendation, they do provide good guidance"*
	Adaptability	Deprescribing paced to patient preferences	*"… allows the patient to go at their own pace."*	Embedding deprescribing tools into existing systems and workflows	*"Integration with the EMR and/or digital Medication record"*
	Trialability	Ability to safely trial deprescribing	Individual response is not to be published	-	-
	Design Quality and Packaging	-	-	Graphics and flowcharts; succinct, layered information; and searchable by medicine.	*"… it would be useful and used if in a suitable layout on a website, see for example eTG or even AMH online. Needs to be searchable, succint info with option to read more details."*
	Cost	Government-funded medication review	*"I now have a Federally sponsored pharmacist home visit a couple of times a year."*	Minimal out-of-pocket costs	*"...and not having to pay high fees."*
**Outer setting**	External Policy & Incentives	External organisation endorsements	*"RACGP, and the AMA etc need to be on board"*	Non-pharmacological alternatives	*"Acceptance of, availability and timely access to non-pharmacological alternatives"*
**Inner setting**	Structural characteristics	Regular clinician access	*"Having regular access to medical practitioner"*	-	-
	Networks & Communications	Communication that is honest, empathetic, attentive, respectful, person-	*"The manner in which the question of deprescribing was raised would be significant. For example, a patient, respectful, unhurried approach and a willingness to repeat information when*	Multidisciplinary communication	*"Improved communication between GPs, specialists, hospital-based clinicians and community pharmacists"*

		centred, and collaborative	*necessary should ensure effective communication."*		
	Culture	Prudent, patient-centred prescribing culture prioritising well-being, genuine care, and treating deprescribing as a routine process	*"Patient-centered care, prescriber supports patient goals rather than trying to choose for the patient. Prescriber understands why linear tapering is usually inferior to hyperbolic tapering, and that withdrawal reactions are often mistaken for relapse. Prescriber has experience with acute, post-acute, and protracted withdrawal effects. Prescriber does not use biomedical model to pathologize patient's withdrawal symptoms as an underlying mental disorder or does not pathologize patient's disagreements with prescriber."*	-	-
	Implementation climate	Long-term or trusting relationship with a clinician	*"Familiarity with your providers and trusting them"*	Shared decision making and good relationship with clinicians	*"Having a good relationship with your provider"*
	Tension to change	Open-minded and willing to learn	*"A provider who has … an open mind"*	-	-
	Available resources	Adequate time and full clinical profile	*"Having latest documentation (i.e. blood test results & other reports like x-ray reports & images).*<br>*these usually indicate status quo upon which to base further recommendations."*	Longer consultation time	*"Not feeling rushed at appointments"*
	Access to knowledge & information	Education, training, and access to resources about deprescribing	*"Dissemination of this material to healthcare providers."*	Education, training, and access to resources about deprescribing	*"The ease of reference to the synthesised evidence within the guidelines appears to be a great facilitator to evidence-based clinical practice, however we do not work within clinical practice so are unable to comment."*

**Characteristics of Individuals**	Knowledge & Beliefs about the Intervention	Aware/informed of the underlying principles or rationale for deprescribing and continuation of medicines	*"If they have plenty of awareness and exposure to the benefits of deprescription and understand the extended follow-up requirements and continuity of treatment."*	Aware/informed of the underlying principles or rationale for deprescribing and continuation of medicines	*"Healthcare professions knowledge of deprescribing …"*
	Self-efficacy	Lifestyle changes, past successes and encouragements	*"… I have some confidence having been successful twice."*	Confidence	*"Healthcare professionals confidence with shared decision-making."*
	Individual Stage of Change	Interest in deprescribing	Individual response is not to be published	-	-
**Process**	Planning	Having a clear and collaborative plan	*"Having a Care Plan."*	-	-
	Champions	Support systems (Carer, advocate, family member, relatives, speaker, letter of support, support groups)	*"Yes. Deprescribing can be confusing and difficult. Mentally and physically. There needs to be a support system in place for the patient and or the caregiver."*	Community support	*"… They need community because tapering is such a strange experience …"*
	Key Stakeholders	Multidisciplinary approach (pharmacists, nurses, psychologists, specialist)	*"I feel it needs a team approach. Inclusion of the patient pharmacist as part of the consult would be very powerful. Perhaps the practice nurse Pharmacist expertise and personal knowledge of the patient is overlooked in the system."*	Multidisciplinary approach (specialists, pharmacists, psychologists)	*"I would ideally get a pharmacist to do HMR review as part of the process, this helps engage the patient in the process."*
	Reflecting & evaluating	Monitoring	*"…my GP suggested I check my eyes for the effect of long-term use of …"*	-	-

Facilitators such as professional endorsement of guidelines, openness to change, positive relationships with clinicians, and shared decision-making corresponded with the "develop stakeholder interrelationships" cluster. Reported needs for clear information, education, and awareness of deprescribing principles aligned with the "train and educate stakeholders" cluster. Facilitators related to deprescribing monitoring and improving self-efficacy for deprescribing corresponded with the "engage consumers" cluster. Other facilitators related to adaptability and trialability of deprescribing aligned with the "adapt and tailor to context" and "use evaluative and iterative strategies" clusters. Facilitators such as practice endorsement from external organisations and availability of non-pharmacological alternatives corresponded with the "change infrastructure" cluster. Finally, facilitators such as multidisciplinary involvement, sufficient consultation time, costs, and complete clinical information aligned with strategies within "utilise financial strategies" and "support clinicians".

Table S5. Mapping of determinants to the Consolidated Framework for Implementation Research (CFIR) domains and constructs

CFIR Domain	CFIR Constructs	Barrier	Facilitator
Intervention characteristics	Evidence Strength & Quality	✓	✓
	Relative advantage	✓	
	Adaptability	✓	✓
	Trialability	✓	✓
	Design Quality and Packaging	✓	✓
	Cost	✓	✓
	Complexity	✓	
Outer setting	External Policy & Incentives	✓	✓
	Patient needs and resources	✓	
Inner setting	Structural characteristics	✓	✓
	Compatibility	✓	
	Networks & Communications	✓	✓
	Culture	✓	✓
	Implementation climate	✓	✓
	Tension to change	✓	✓
	Organisational incentives and rewards	✓	
	Leadership engagement	✓	
	Available resources	✓	✓
	Access to knowledge & information	✓	✓
Characteristics of Individuals	Knowledge & Beliefs about the Intervention	✓	✓
	Self-efficacy	✓	✓
	Individual Stage of Change	✓	✓
Process	Planning		✓
	Champions		✓
	Key Stakeholders		✓
	Reflecting & evaluating		✓
