# Supplementary material for: A qualitative analysis to identify implementation strategies for a deprescribing clinical practice guideline using the CFIR-ERIC Matching Tool: perspectives from consumers and professional stakeholders
Source: Int J Clin Pharm. 2026 Sep 3;48(6):2403–17. doi: 10.1007/s11096-026-02218-9 (PMC13731450; doi:10.1007/s11096-026-02218-9)
Supplement: Supplementary file 3 [file 11096_2026_2218_MOESM3_ESM.pdf]

THE UNIVERSITY OF WESTERN AUSTRALIA

PICF

Key information

- To access the DRAFT Guidelines and all related DRAFT documents, please visit 🔗 deprescribing.com/public-consultation. Scroll to the bottom of the page > under Public Consultation Materials > Click 'Download'.
- The survey automatically saves your progress, so you can return later if needed. However, you must use the same device and browser to see your previous saved responses.
- You do not have to provide feedback on all sections or respond to all feedback questions in this form. Please focus on the topics of most interest to you and/or your organisation.
- For all other general enquiries, please email 📩 deprescribing@uwa.edu.au (please note that we will not be responding to any feedback on the draft guidelines through this email).
- Submissions close Saturday, **31st May 2025** (11:59 pm AEST). Late submissions will not be accepted.

Participant Information Sheet/ Consent Form

Title: Deprescribing in older people: A clinical practice guideline

Invitation: You are invited to provide feedback on the draft guideline as part of a public consultation process.

What are we asking? We have developed guidelines to provide healthcare professionals with guidance for deprescribing medicines. The guideline states recommendations that are intended to be used within a shared decision-making framework, rather than prescriptive mandates. We are seeking your feedback on the draft guideline, as we value the diverse perspectives, experiences, and knowledge of all stakeholders and community members.

What does participation involve? You will be asked to review the draft guideline and respond to a set of questions in an online survey. The survey will take about 15 minutes to complete, however, you can take as long as you want to answer the questions.

What will be done with your data upon completion? The information contained in your submission will be used to inform further development of the draft clinical practice guideline for deprescribing in older people. All responses will be analysed, and the findings will be published alongside the final guideline. Your responses will remain confidential, and your identity will not be linked to any of the feedback you provide unless you provide consent. Please note that responses may be moderated to remove content

that is inappropriate/offensive or contains sensitive information.

Voluntary Participation and Withdrawal from the Study: Participation is entirely voluntary and withdrawal from the study is allowed at any time. However, due to the nature of the study, any response you provided in the previous rounds will be retained and included for consideration and analysis. There will be no consequences for participation (or non-participation) in the study.

Your Privacy: With your consent, we will acknowledge your contribution in the final publication. However, if you prefer to remain anonymous, we will ensure your identity remains confidential. All study data will be stored securely for a minimum of 7 years. At the end of the survey, after you have had the chance to review the questions and provide your responses, you will be asked about your preference regarding the publication of your survey submission.

Possible Benefits: Your contribution will help expand resources for deprescribing decision-making, particularly for older people, potentially improving their health outcomes. Although you may not personally benefit directly, your feedback will inform a guideline that could positively impact many people.

Possible Risks and Risk Management Plan: Participants may be inconvenienced by the time required to take part in the survey.

Access to results of the study: If you would like to receive the final clinical practice guideline, you may provide your contact details, and we will notify you when it is available.

Contacts: If you would like to participate or discuss any aspect of this study please feel free to contact Ms Amanda Quek (amanda.quek@research.uwa.edu.au) or Dr Amy Page (amy.page@uwa.edu.au). Approval to conduct this research has been provided by the University of Western Australia, in accordance with its ethics review and approval procedures. Any person considering participation in this research project, or agreeing to participate, may raise any questions or issues with the researchers at any time. In addition, any person not satisfied with the response of researchers may raise ethics issues or concerns, and may make any complaints about this research project by contacting the Human Ethics Office at the University of Western Australia at (08) 6488 3703 or by emailing humanethics@uwa.edu.au. All research participants are entitled to retain a copy of any Participant Information Form and/or Participant Consent Form relating to this research project.

Consent statement:
By completing this survey, I acknowledge that:
• I have read the information provided, and any questions I asked have been answered to my satisfaction
• I agree to provide feedback on the guidelines, realising that I may stop at any time without reason and without prejudice
• I understand that the giving of my consent is entirely voluntary
• I understand that all identifiable information that I provide is treated as strictly confidential and will not be released by the investigator in any form that may identify me. The only exception to this principle of confidentiality is if this information is required by law to be released
• I have been advised as to what data is being collected, the purpose for collecting the data, and what will be done with the data upon completion of the research. I agree that research data gathered for the study may be published provided my name or other identifying information is not used (unless I have consented to it).

Yes

No

Demographics, CoI

About You

What is your full name?

Please note your preference for publication of your survey response will be asked at the end of the survey.

What is your email address?

This is so we can contact you for clarification if needed. Please note that your email address will not be published.

What is your postcode?

What is your age?

20-29

30-39

40-49

50-59

60-69

70+

What is your gender identity?

Male

Female

Non-binary

Prefer not to say

Other (please specify)

Do you identify with any of the following?

Aboriginal and Torres Strait Islander peoples

People living in a rural or remote area

LGBTQIA+

People from culturally and linguistically diverse communities

None of the above

Prefer not to say

How did you find out about this guideline consultation?

Newspaper advertisement

Social media (e.g., Facebook, Twitter, LinkedIn)

Email from an organisation or professional group

Word of mouth (e.g., friend, colleague)

Other (please specify)

Please provide details of the conflict of interest/s
If not applicable, please enter 'N/A' in the box below.

What is your interest in this topic on deprescribing?

I am a consumer with lived or living experience

I have a family member/relative or care for someone taking long-term medication(s)

Consumer advocate (please provide more information)

I have a work-related interest in deprescribing (please provide more information)

I am a member of the public with a general interest (please provide more information)

I am a health professional – Primary care (please specify your area of specialty)

I am a health professional – Specialist (please specify your area of specialty)

I am a health professional – Allied health (please specify your area of specialty)

I am a health professional – Other healthcare provider (please specify your area of specialty)

Health policy maker /service provider (please provide more information)

Researcher/ academic (please provide more information)

Others (please specify)

Are you participating in this consultation as a representative of a group or an organisation?

Yes

No

What is the name of the organisation/group you are representing?

What is your job title?

Medicine question

Are you or the person you care for currently taking medicines, including prescription medicines, over-the-counter medicines, vitamins, and supplements?

Yes

No

How many medicines are you or the person you care for currently taking? (this includes prescription medicines, over-the-counter medicines, vitamins, and supplements)

**Please enter a numeric answer, i.e. "5"*

Medications used

Please list the medicine you or the person you care for are currently taking, including prescription medicines, over-the-counter medicines, vitamins, and supplements.

What are you or the person you care taking this medicine for?

5 Feedback Questions

Feedback

In your view, is there a need for this guideline that provides broad guidance on deprescribing medications and is intended to support shared decision-making with individuals, their family members, carers, and/or support persons?

Please explain your answer.

Is the guideline clear and easy to understand? If not, which sections need improvement?

Are there any terms or concepts that require further clarification?

Does the guideline address the key concerns relevant to your role?

Does the guideline provide sufficient detail to support decision-making for deprescribing? If not, what additional information would be helpful?

Barriers/Facilitators

Finally, we would like to ask you a few final questions about how you would see this guideline being used in practice.

Implementation Questions

(If relevant, consumers/carers, member of the public with general interest, consumer advocacy) Would you require any additional support to discuss deprescribing with your healthcare providers?

(If relevant, consumers/carers, member of the public with general interest, consumer advocacy)
What barriers do you think you would encounter in discussing deprescribing with your healthcare providers?

(If relevant, consumers/carers, member of the public with general interest, consumer advocacy)
What makes it easier to discuss deprescribing with your healthcare providers?

(If relevant, healthcare professionals, researchers, health policy/management)
Would you require any additional resources or tools to implement this guideline in practice?

(If relevant, healthcare professionals, researchers, health policy/management)
What barriers do you think people would encounter in implementing the guideline?

(If relevant, healthcare professionals, researchers, health policy/management)
Are there any facilitators of using this guideline in clinical practice?

To what extent would this guideline fill your needs? If not, why not? What additions and changes are required?

Please be as specific as possible in your response.

Preference for publication

Preference for publication

What is your preference for publication of your individual survey submission?

Responses to this consultation may be included in a report. Responses may be published in full (verbatim) should you give your permission. Aggregated responses may be used to inform the guidelines. Please indicate your preference for the publication of your survey submission:

Publish response, including my name only

Publish response, including my orgnisation's name only

Publish response, including both my name and my organisation's name

Publish response without my name or my organisation's name

My individual submission is not to be published in full and may only be used to inform the guidelines, reports or research publications

Powered by Qualtrics
